# Novel Human Bocavirus in Children with Acute Respiratory Tract Infection

**DOI:** 10.3201/eid1602.090553

**Published:** 2010-02

**Authors:** Jing-rong Song, Yu Jin, Zhi-ping Xie, Han-chun Gao, Ni-guang Xiao, Wei-xia Chen, Zi-qian Xu, Kun-long Yan, Yang Zhao, Yun-de Hou, Zhao-jun Duan

**Affiliations:** First Hospital of Lanzhou University, Lanzhou, People’s Republic of China (J-R. Song, Y. Jin, W-X. Chen, K-L. Yan, Y. Zhao); China Center for Disease Control and Prevention, Beijing, PRC (J-R. Song, Y. Jin, Z-P. Xie, H-C. Gao, N-G. Xiao, W-X. Chen, Z-Q. Xu, K-L. Yan, Y. Zhao, Y-D. Hou, Z-J. Duan); 1 These authors contributed equally to this article.

**Keywords:** Human bocavirus, children, acute respiratory tract infection, viruses, respiratory infections, dispatch

## Abstract

Human bocavirus (HBoV) and HBoV2, two human bocavirus species, were found in 18 and 10 of 235 nasopharyngeal aspirates, respectively, from children hospitalized with acute respiratory tract infection. Our results suggest that, like HBoV, HBoV2 is distributed worldwide and may be associated with respiratory and enteric diseases.

Acute respiratory tract infection (ARTI) is a leading cause of illness and death in infants and young children (*1*). Human bocavirus (HBoV) was first identified in respiratory samples from children and was proposed to be pathogenic in humans (*2*). Subsequently, HBoV infections were reported in children with ARTI (*1**,**3**,**4*), and found in stool samples from children with gastroenteritis worldwide (*5*). In 2009, Kapoor et al. identified another human parvovirus that was most closely related to HBoV and named it HBoV genotype 2 (HBoV2) (*6*). In this study we examined its presence in nasopharyngeal aspirates (NPAs) from children hospitalized with ARTI.

## The Study

NPA samples were collected from 235 children hospitalized with ARTI at the First Hospital of Lanzhou University, Gansu Province, China during December 2007–November 2008. All patients were <15 years of age, and informed consent was obtained from their parents. Demographic data and clinical findings were recorded. The study protocol was approved by the hospital ethics committee.

DNA and RNA were extracted from the NPAs by using QIAamp DNA and QIAamp viral RNA mini kits (QIAGEN, Beijing, China). The cDNA sample was synthesized by using random hexamer primers. A standard reverse transcription–PCR was used to screen for human rhinovirus, respiratory synctial virus (RSV), influenza virus A, influenza virus B, parainfluenza virus 1–3, human metapneumovirus, human coronavirus (HCoV)–NL63, and HCoV-HKU1, and PCR method was used to screen for adenovirus (ADV) (*7**–**11*).To screen for HBoV, PCR was performed by using primers 188F and 542R, as described by Allander et al. (*2*). For HBoV2, nested PCR was performed with sf1/sr1 and sf2/sr2 primers, which amplified a 455-bp fragment of the partial NS1 gene, as described (*6*).

In addition, we designed HBoV2 forward (SN1: 5′-ACCAGTGGGAGAACCACAAG-3′) and reverse (SN2: 5′-GGCATTTGTTTCCATGCTTT-3′) primers, which produced a 563-bp fragment of the NP1 gene of HBoV2. Positive and negative controls were included for each PCR. Purified PCR products were sequenced by using SinoGenoMax. ClustalX (ftp://ftp-igbmc.u-strasbg.fr/pub/ClustalX) was used to align the obtained sequences with sequences available in GenBank.

In total, 260 viruses were identified in 196 (83.4%) of the 235 children. Using nested PCR, we found 21 positive specimens; further nucleotide sequence analysis showed that 10 (4.3%) were HBoV2 and 11 were HBoV (Figure, panel A). All 11 HBoV strains detected by using HBoV2 nested-PCR were included in the 18 HBoV-positive patients as determined by PCR using primers 188F and 542R. Of the 10 HBoV2-positive patients, 7 (70%) were co-infected with other respiratory viruses, including 4 patients with RSV. Of the 18 HBoV-positive patients, 12 (66.7%) displayed co-infections. There were no statistically significant differences in the HBoV2 and HBoV detection (p = 0.119 by χ^2^ test) and co-infection (p = 1.000 by Fisher exact test) rates.

**Figure:**
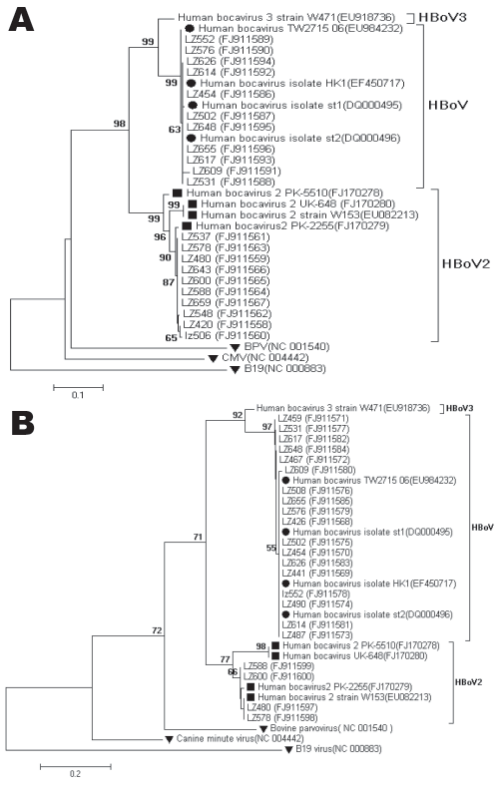
Phylogenetic analysis of A) the partial nonstructural protein 1 (NS1) nucleotide sequences (412 bp) and B) the partial nucleoprotein 1 (NP1) nucleotide sequences (256 bp) of human bocavirus 2 (HBoV2), Gansu Province, People’s Republic of China. The phylogenetic trees were constructed by the neighbor-joining method using MEGA 3.1 (www.megasoftware.net), and bootstrap values were determined for 1,000 replicates. Bootstrap values >50% are shown at the branching points. Human bocavirus (HBoV) and HBoV2 reference sequences are indicated by circles and squares, respectively. Bovine parvovirus (BPV), cytomegalovirus (CMV), and human parvovirus B19 (B19) reference sequences are indicated by inverted triangles. Scale bars indicate nucleotide substitutions per site. Reference sequences were obtained from GenBank (accession nos. DQ000495, DQ000496, EF450717, EU082213, EU918736, EU984232, FJ170278, FJ170279, FJ170280, NC001540, NC004442, and NC000883). Sequences generated in this study were deposited in GenBank under accession nos. FJ911558–FJ911600.

Of the 10 HBoV2-positive patients, 9 were male and 1 was female (χ^2^ = 1.957, p = 0.162). The median age was 8.5 months, and 9/10 (90%) were <3 years old. HBoV2 infections were detected throughout the year. Of the 18 patients who were HBoV positive, 11 were male and 7 were female (χ^2^ = 0.084, p = 0.772). The median age of patients was 11.5 months, and 16/18 (88.9%) were <3 years old.

HBoV infections were detected in every month except August, with peaks in December (3 cases) and January (4 cases). The main diagnoses of the 3 patients with HBoV2 monoinfection were acute asthmatic bronchopneumonia, bronchopneumonia, and acute upper respiratory tract infection in 1 patient each. For the 6 patients with HBoV monoinfection, the main diagnoses were acute asthmatic bronchopneumonia (4 cases) and bronchopneumonia (2 cases). The clinical signs and symptoms of HBoV2 and HBoV positive patients included cough, fever, sputum production, crackles, wheezing, rhinorrhea, cyanosis, vomiting, and diarrhea (Table 1). For patients with HBoV2 monoinfection, the median hospital stay was 11.3 days (range 4–23 days), and 2 had underlying illnesses (idiopathic pulmonary hemosiderosis and iron deficiency anemia). The chest radiograph of 1 patient showed upper middle zone air-space shadows. For patients with HBoV monoinfection, the median hospital stay was 7.8 days (range 6–10 days), and none had underlying illnesses. Chest radiographs of 2 patients showed shadows in the left lung zone.

**Table 1:** Comparison of clinical characteristics among different groups of children with acute respiratory infections, Gansu Province, China*

Characteristic	Group 1, n = 3	Group 2, n = 7	Group 3, n = 6	Group 4, n = 12	p values		
					Group 1 vs. Group 2	Group 3 vs. Group 4	Group 1 vs. Group 3
Male sex	3 (100)	6 (85.7)	3 (50)	8 (66.7)	0.499†	0.145†	1.000†
Age <3 y	3 (100)	6 (85.7)	6 (100)	10 (83.3)	1.000†	0.307†	1.000†
Median duration of hospitalization, d (range)	11.3 (4–23)	9 (8–11)	7.8 (6–10)	10.3 (5–20)	0.492†	0.187‡	1.000‡
No. underlying diseases	2	1	0	1	0.183†		
Clinical diagnosis							
AURI	1 (33.3)	0	0	1 (8.3)			
Suppurative tonsillitis	0	0	0	1(8.3)			
Acute asthmatic bronchopneumonia	1 (33.3)	4 (57.1)	4 (66.7)	7 (58.3)	0.500§	0.572§	0.405§
Bronchopneumonia	1 (33.3)	1 (14.3)	2 (33.3)	3 (25.0)	0.533§	0.561§	0.774§
Acute bronchitis	0	2 (28.6)	0	0			
Clinical signs							
Fever	2 (66.7)	5 (71.4)	2 (33.3)	7 (58.3)	0.708§	0.310§	0.405§
Cough	2 (66.7)	5 (71.4)	5 (83.3)	10 (83.3)	0.708§	0.730§	0.583§
Wheeze	1 (33.3)	4 (57.1)	4 (66.7)	7 (58.3)	1.000§	0.572§	0.405§
Rhinorrhea	2 (66.7)	2 (28.6)	2 (33.3)	5 (41.7)	0.500§	0.572§	0.405§
Sputum production	1 (33.3)	5 (71.4)	5 (83.3)	4 (33.3)	0.500§	0.066§	0.226§
Crackles	2 (66.7)	6 (85.7)	6 (100)	7 (58.3)	1.000§		
Vomiting	0	1 (14.3)	1 (16.7)	0			
Diarrhea	2 (66.7)	1 (14.3)	1 (16.7)	1 (8.3)	0.183§	0.569§	0.226§

*Values are no. (%) children except as indicated. HBoV, human bocavirus; AURI, acute upper respiratory tract infection. Group 1, HBoV2 monoinfection; Group 2, HBoV2 co-infection; Group 3, HBoV1 monoinfection; Group 4, HBoV1 co-infection. †By χ^2^ test. ‡By Mann-Whitney test. §By Fisher exact test.

Ten HBoV2 NS-1 sequences (455 bp) shared 98%–99% and 95%–96% nucleotide sequence identity and 99%–100% and 98%–99% deduced amino acid sequence identity with HBoV2 strain PK-2255 (FJ170279) and HBoV2 strain W153 (EU082213), respectively; These sequences also shared 81%–82% and 83.3%–84.4% nucleotide sequence identity and 90% and 88% deduced amino acid sequence identity with the HBoV prototype strain ST1 or ST2 (DQ000495 and DQ000496) and human bocavirus 3 strain W471 (EU918736), respectively. The 4 HBoV2 NP-1 sequences shared 98%–99% and 97.6%–98.3% nucleotide sequence identity and 98%–100% and 97%–100% deduced amino acid sequence identity with HBoV2 strain PK-2255 and HBoV2 strain W153, respectively, and shared 74%–78% and 69.7%–70.3% nucleotide sequence identity and 69%–73% and 58%–62% deduced amino acid sequence identity with the prototype strain ST1 or ST2 and human bocavirus 3 strain W471, respectively. The nucleotide and deduced amino acid sequences of NS-1 and NP-1 shared high identities (>97%) with the HBoV2 and HBoV sequences (Table 2). Phylogenetic analysis indicated that HBoV2 is more closely related to HBoV (Figure).

**Table 2:** Nucleotide and amino acid sequence comparisons among and between human bocaviruses, Gansu Province, China*

Virus comparison	NS1 sequence identity, %			NP1 sequence identity, %	
	Nucleotide	Amino acid		Nucleotide	Amino acid
With HBoV2 PK-2255 sequence	98–99	99–100		98–99	98–100
With HBoV2 strain W153 sequence	95–96	98–99		97.6–98.3	97–100
With the HBoV prototype strain ST1 or ST2 sequence	81–82	90		74–78	69–73
With HBoV 3 strain W471 sequence	83.3–84.4	88		69.7–70.3	58–62
Among the HBoV2 sequence	97–99	98		98–100	99–100
Among the HBoV sequence	97–98	99		97–100	98–100

*HBoV, human bocavirus; NS1, nonstructural protein 1; NP1, nucleoprotein 1.

## Conclusions

Using nested PCR and sequencing, we identified HBoV2 infections in 10 (4.3%) of 235 NPAs from children hospitalized with ARTI. Most of the patients were <3 years old. In HBoV2-positive patients, co-infection was high (70%), with RSV being the most common co-pathogen. Primers SN1 and SN2 were designed to detect the NP1 gene in the 10 HBoV2-positive patients. However, only 4 gave positive results, which occurred because of the low PCR sensitivity with this pair of primers and because the NP1 gene of HBoV2 is divergent, as described (*6*). Furthermore, as previous studies (*6*,*12*) pointed out, potential recombination upstream from the NP1 gene may explain the lower detection. Phylogenetic analysis showed that the NS-1 region of the HBoV2 strain (LZ480 and LZ578) clustered closely with that of the HBoV2 PK-2255 strain (FJ170279), and the NP-1 region clustered closely with that of the HBoV2 W153 strain (EU082213), suggesting potential recombination in the HBoV2 strains (Figure). In addition, 11 HBoV sequences were amplified by using nested-PCR for HBoV2. In the future, HBoV2-specific primers should be designed to investigate the prevalence of HBoV2 and its potential association with disease.

We found no difference in the clinical symptoms or length of hospital stay between the groups with HBoV2 and HBoV monoinfection, as well as between the groups with HBoV2 monoinfection and HBoV2 co-infection (Table 1). Statistical analysis indicated that HBoV2 and HBoV co-infection obviously did not correlate with disease severity (data not shown). Two of 3 patients with HBoV2 monoinfection had diarrhea with no vomiting (Table 1), and only 1 of 10 patients who were HBoV2 positive vomited. Further investigation is needed to exclude oral or inhaled gastric viruses as possible sources of NPA-associated HBoV2. Phylogenetic analysis showed a high degree of similarity between HBoV2 sequences found in China and those in other areas (Figure). Our results suggest that like HBoV, HBoV2 is distributed worldwide and may be associated with respiratory and enteric diseases. Additional studies are needed to confirm the association between human bocavirus species (HBoV2 and HBoV) and respiratory tract infections or other diseases.
